# Supplementary figures and images for: Green Extracts and UPLC-TQS-MS/MS Profiling of Flavonoids from Mexican Oregano (Lippia graveolens) Using Natural Deep Eutectic Solvents/Ultrasound-Assisted and Supercritical Fluids
Source: Plants (Basel). 2023 Apr 18;12(8):1692. doi: 10.3390/plants12081692 (PMC10145289; doi:10.3390/plants12081692)

**Figure S1. UPLC-MS chromatograms for flavonoid standards.**

**Flavonoids**

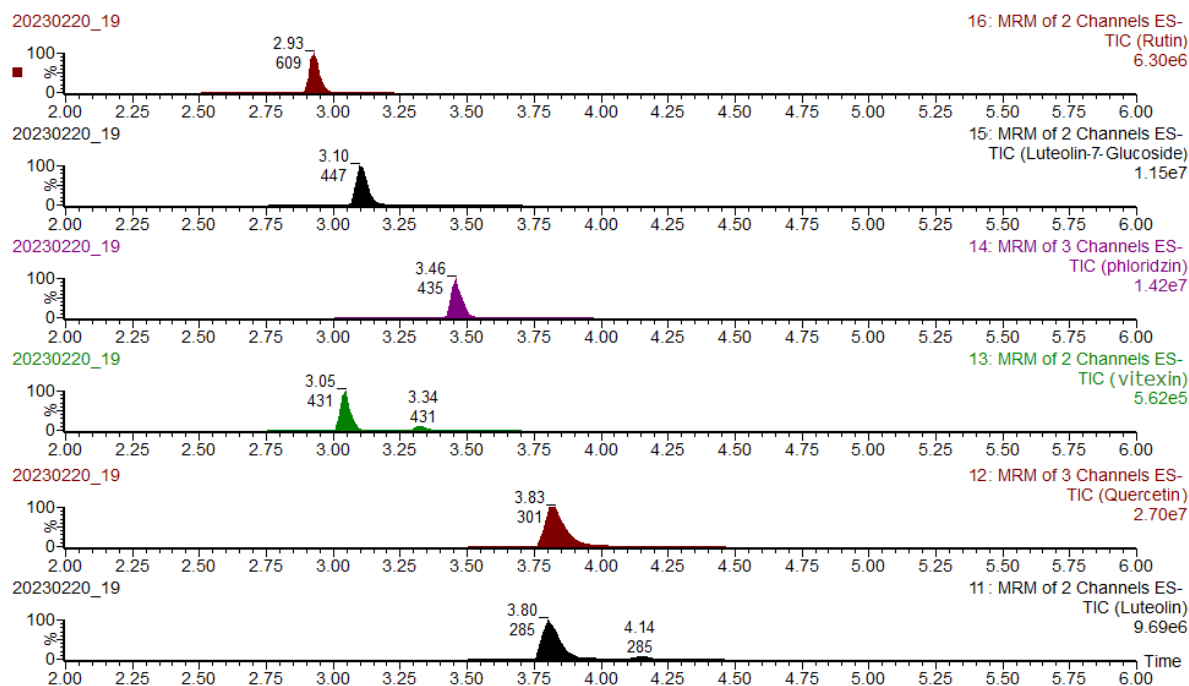

**Flavonoids**

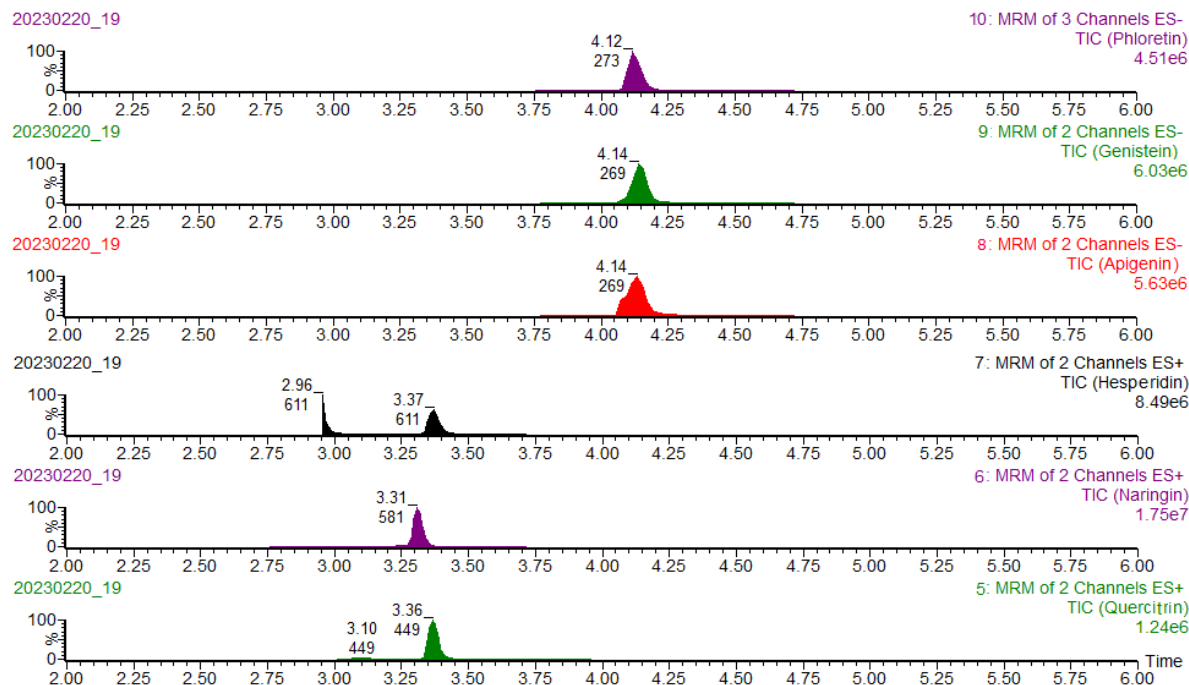

# Flavonoids

20230220\_19

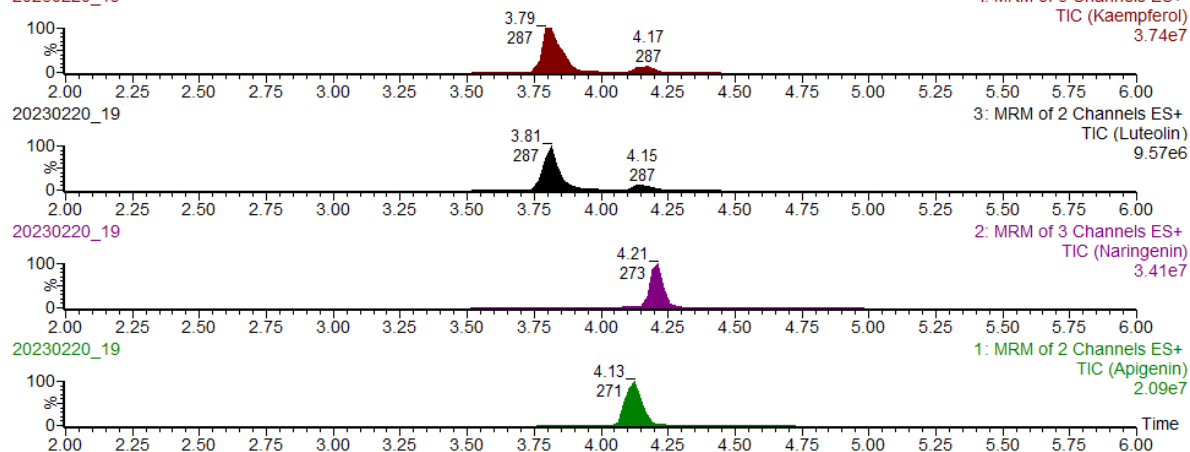

Supplement: Supplementary file 1 [file plants-12-01692-s001.zip › plants-2325554-supplementary.pdf]
